# Profile and outcome of patients with upper gastrointestinal bleeding presenting to urban emergency departments of tertiary hospitals in Tanzania

**DOI:** 10.1186/s12876-019-1131-9

**Published:** 2019-12-10

**Authors:** Shaffin S. Rajan, Hendry R. Sawe, Asha J. Iyullu, Dereck A. Kaale, Nancy A. Olambo, Juma A. Mfinanga, Ellen J. Weber

**Affiliations:** 1 Emergency Medicine Department, Muhimbili University of Health and Allied Science, P.O. Box 65001, Dar es Salaam, Tanzania; 2 Emergency Medicine Department, Muhimbili National Hospital, Dar es Salaam, Tanzania; 3 Department of Emergency Medicine, University of California, San Francisco, CA USA; 4 Critical Care Unit, Regency Medical Centre, Dar es Salaam, Tanzania

**Keywords:** Upper gastrointestinal bleeding, Non-traumatic patients, Emergency department, Tanzania, Sub Saharan Africa.

## Abstract

**Background:**

Upper gastrointestinal bleeding (UGIB) is a common emergency department (ED) presentation with high morbidity and mortality. There is a paucity of data on the profile and outcome of patients who present with UGIB to EDs, especially within limited resource settings where emergency medicine is a new specialty. We aim to describe the patient profile, clinical severity and outcomes of the patients who present with UGIB to the ED of tertiary referral hospitals in Tanzania.

**Methods:**

This was a prospective cohort study of consecutive adult (≥18 years) patients presenting to the EDs of Muhimbili National Hospital (ED-MNH) and MUHAS Academic Medical Centre (ED-MAMC), in Tanzania with non-traumatic upper gastrointestinal bleeding (UGIB) from July 2018 to December 2018. Patient demographic data, clinical presentation, and ED and hospital management provided were recorded. We used the clinical Rockall score to assess disease severity. The primary outcome of 7- day mortality was summarized using descriptive statistics. Regression analysis was performed to identify predictors of mortality.

**Results:**

During the study period, 123 patients presented to one of the two EDs with an UGIB. The median age was 42 years (Interquartile range (IQR) 32–64 years), and 87 (70.7%) were male. Hematemesis with melena was the most frequently encountered ED complaint 39 (31.7%). Within 7 days, 23 (18.7%) patients died and one-third 8 (34.8%) of these died within 24 h. There were no ED deaths. About 65.1% of the patients had severe anemia but only 60 (48.8%) received blood transfusion in the ED. Amongst those with history of (h/o) esophageal varices 7(41.2%) did not receive octreotide. Upper GI endoscopy, was performed on 46 (37.4%) patients, of whom only 8 (17.4%) received endoscopy within 24 h (early UGI endoscopy). All patients who received early UGI endoscopy had a low or moderate clinical Rockall score i.e. < 3 and 3–4. No patient with scores of > 4 received early UGI endoscopy. Age > 40 years was a significant independent predictor of mortality (OR = 7.00 (95% CI 1.7–29.2). Having a high clinical Rockall score of ≥ 4 was a significant independent predictor of mortality (OR = 6.4 (95% CI 1.8–22.8).

**Conclusions:**

In this urban ED in Sub-Saharan Africa, UGIB carried a high mortality rate. Age > 40 years and clinical Rockall score ≥ 4 were independent predictors of higher mortality. Future studies should focus on evaluating how to improve access to UGI endoscopy so as to improve outcomes.

## Background

Upper Gastrointestinal bleeding (UGIB) is a medico-surgical emergency. Although there has been a global decline in the mortality associated with UGIB, the incidence and mortality associated with GI bleeding remains high in limited income countries. In the United States, UGIB accounts for 300,000 admissions per year with approximately 5% mortality rate [1], whereas in several studies from sub-Saharan Africa, mortality ranges from 6 to 30% [2–5]. It is not clear if the higher mortality seen in low and middle income countries (LIMC’s) is due to patient demographics, severity on presentation, etiology or compliance with care standards. Esophageal varices has been implicated as the most common cause of UGIB in several African studies [2–11]. Rather than being due to alcoholic liver disease as in high income countries (HIC’s), varices in sub-Saharan Africa result from Schistosoma-related portal hypertension [5, 8, 12]. This is in contrast to HIC where erosive gastritis has been commonly implicated [1, 13, 14]. Other potential contributors to the higher mortality may be the severity of disease presentation, as there is little primary care in these settings, and many patients seek care very late in their disease.

Another potential contributor is failure to treat patients in accordance with management guidelines. Proton pump inhibitor (PPI) for those with suspected Non-variceal UGIB (NVUGIB), somatostatin analogues such as octreotide and antibiotics in suspected cases of variceal UGIB (VUGIB) and in those with clinical suspicion of liver disease, timely blood transfusion and early use of endoscopy may not occur in these settings due to lack of appropriate specialists, lack of resources or supply chain issues [15–17]. A knowledge gap on the current UGIB management guidelines and recommendations amongst the health care providers may also contribute to inadequate management of UGIB cases. Prior studies in Tanzania studies demonstrate lack of appropriate care offered to patients with UGIB. Nearly half of the patients in one the studies did not receive endoscopic evaluation and treatment [5] whereas 47.1% of the patients who required blood transfusion, did not receive blood transfusion in another study [10].

There is little information on the presentation, etiology and management of patients presenting to EDs and this is particularly true for LIMCS, where emergency medicine is a new specialty. The presence of full capacity emergency departments at Muhimbili National Hospital (MNH) and MUHAS Academic Medical Centre (MAMC) in Dar es Salaam have provided the opportunity for early stabilization and management of patients presenting with UGIB. The aim of this study was to describe the patient profile, clinical severity and outcomes of the patients who present with UGIB to the ED.

## Methods

### Study design

This was a prospective cohort study of adult patients presenting to two EDs in Tanzania with upper gastrointestinal bleeding from July 2018 to December 2018.

### Study setting

This study was conducted at the ED- MNH and ED-MAMC, Dar es Salaam Tanzania. MNH is a public, tertiary referral hospital which opened in 2010. It is the site of the first full capacity public ED in Tanzania and the primary training site for the only Emergency Medicine (EM) residency program in the country. MAMC is a recently inaugurated full capacity university health facility with a state of the art emergency medicine department, located approximately 30 km from MNH. MNH and MAMC both have a fully equipped endoscopy unit.

### Study participants

All consenting adults (age greater than or equal to 18 years) presenting with UGIB unrelated to a recent trauma were eligible for the study. We excluded patients who had previously been enrolled in the study who presented with recurrent episodes of UGIB during the period of the study.

### Study protocol

Research assistants were scheduled to collect data 24 h a day, seven days a week and during that time patients were consecutively approached and asked for consent to be followed for the study. Demographics, clinical presentation, initial management, and ED outcomes were collected by the research assistant using information given by the patient or caregiver, the treating physician, and data found in the electronic medical record (Wellsoft™). A structured case report form was used to record all participants’ information. All admitted patients were followed up in a hospital ward and if discharged through mobile phone calls to determine their outcome from the EMD-MNH and EMD-MAMC, at 24-h and 7-days.

### Assessment of disease severity

Clinical severity was measured using the pre-endoscopic Rockall score (Table 1) and the Glasgow-Blatchford (Table 2). For pre-endoscopic Rockall score, scores < 3 signifies low risk, scores 3–4 signifies moderate risk and score > 4 signifies high risk for re-bleeding, mortality or surgery [19]. For Glasgow-Blatchford score, a score of < 3 is considered Low-risk, whereas a score greater than ≥ 3 is high risk, thus needing intervention, transfusion, endoscopy or surgery. The cut-off value for GBS severity determined as per the study by Ramfrez et al [20].

**Table 1:** Clinical (Pre-Endoscopic) Rockall Score

COMPONENT	0	1	2	3
AGE (yrs)	< 60	60–79	> 80	
HEMODYNAMICS	HR < 100 SBP > 100	HR > 100 SBP > 100	SBP < 100	
COMORBIDITIES	NONE	–	IHD, CHF, ANY MAJOR COMORBIDITIES	RENAL FAILURE, LIVER FAILURE, METASTASIS

*Source:* Wang2013

Total score is calculated by addition of individual scores

**Table 2:** Glasgow-Blatchford Score

ADMISSION RISK MARKER	SCORE COMPONENT
BLOOD UREA (mmol/L)	
6.5–8	2
8.0–10.0	3
10.0–25.0	4
> 25	6
HEMOGLOBIN FOR MEN(g/dl)	
12.0–12.9	1
10.0–11.9	3
< 10	6
HEMOGLOBIN FOR WOMEN(g/dl)	
10.0–11.9	1
< 10	6
SYSTOLIC BLOOD PRESSURE (mmHg)	
100–109	1
90–99	2
< 90	3
OTHER MARKERS	
PULSE RATE > 100 bpm	1
PRESENATION WITH MELENA	1
PRESENTATION WITH SYNCOPE	2
HEPATIC DISEASE	2
CARDIAC FAILURE	1
TOTAL	

*Source:* Cheng 2012 [18]

### Emergent and early upper GI endoscopy

Emergent upper GI endoscopy was defined as endoscopy performed within 12 h of ED presentation whereas early upper GI endoscopy was defined as endoscopy performed within 24 h of ED presentation [21].

### Outcomes

The primary outcome was 7 day mortality due to any cause and not only related to GI bleeding. Secondary outcomes were ED and hospital length of stay and 24 h mortality.

### Data analysis

Data from the case report form was entered into Research Electronic Data Capture (REDCap) software (version 7.2.2, Vanderbilt, Nashville, TN, USA) and transferred into the Statistical Package for Social Science (SPSS) (version 25.0, IBM, LTD, North Carolina, USA). Descriptive statistics were computed with continuous variables presented as mean +/− standard deviation (SD) or median with it’s IQR depending on distribution. Categorical variables are expressed as number and percentage. Univariate associations between categorical variables and outcomes were computed using the Pearson Chi-square test. Multivariate regression analysis was completed on variables with *p* value ≤0.20 in the univariate analysis to identify predictors of 7-day mortality due to UGIB. Statistical significance was set at *p*-value < 0.05.

## Results

**Fig. 1:**
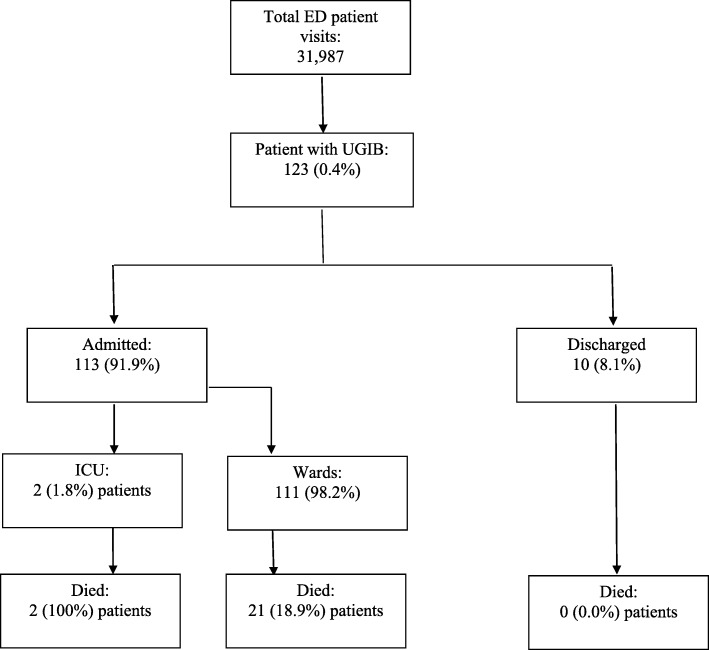
STROBE Flow Diagram

During the period of study, there were 31,987 patient visits (30,800 at EMD-MNH and 1187 at EMD-MAMC). From these we identified 123 (0.4%) patients with upper GI bleeding, all of whom were eligible and consented to be in the study (Fig. 1). Median age was 42 [IQR 32–64] years and 87 (70.7%) were male; 87 (70.7%) were married 44 (35.8%) were self-employed. The majority of patients 77 (62.6%) had been previously seen at a lower capacity health facility and transferred to one of the two study sites. Most patients (99, 80.5%) were uninsured. (Table 3).

**Table 3:** Demographic and Clinical Characteristics of adult patients presenting with UGIB

VARIABLE	FREQUENCY (%)
Age (yrs.)	
Median age	42 years (IQR 32–64)
> 40 years	64 (52.0%)
Sex	
Male	87 (70.7%)
Referral Status	
Hospital referred	77 (62.6%)
Insurance Status	
Uninsured	99 (80.5%)
Presenting Complaint	
Hematemesis	34 (27.6%)
Melena	33 (26.8%)
Hematochezia	11 (8.9%)
Hematemesis and Melena	39 (31.7%)
Hematemesis and Hematochezia	6 (4.9%)
Active Bleeding episode in ED	8 (6.5%)
Vital Signs	
Tachycardia (HR > 100 /min)	56 (45.5%)
Hypotension (SBP < 100 mmHg)	23 (18.7%)
Sp02 < 90%	6 (4.89%)
Medical history	
PUD	39 (40.2%)
UGIB	25 (25.8%)
Esophageal Varices	17 (17.5%)
Liver disease	12 (10.3%)
Others	51 (52.6%)
UGI endoscopy	20 (16.3%)
Band ligation	4 (3.3%)
Clinical Rockall Score (*n* = 123)	
3–4	63 (51.2%)
< 3	52 (42.3%)
> 4	8 (6.5%)
Glasgow-Blatchford Score (*n* = 82)*	
≥ 3	71 (86.6%)
< 3	11 (13.4%)

*Cut-off value for GBS severity determined as per the study by *Ramfrez* et al. [20]

### Clinical presentation

The most frequently encountered presenting complaint was a combination of hematemesis and melena, reported by 39 (31.7%) patients. (Table 3) Nearly half of all patients (56, 45.5%) were tachycardic on arrival. All 123 patients with UGIB were scored for the clinical Rockall score. 63 (51.2%) of the patients had a moderate risk clinical Rockall score of 3–4. A Glasgow-Blatchford score (GBS) could be obtained for 82 patients; this was due to missing of one or more of the point of care (POC)/Lab results required for this scoring system. Of those scored, the majority (71, 86.6%) had GBS score of ≥3, thus characterizing these patients as high risk for adverse events. Hemoglobin (Hb) count was obtained in 106 patients. 69(65.09%) of these patients had a Hb < 8 g/dl, thus categorizing them as severe anemia according to the World Health Organization (WHO) classification.

### Management strategies at the emergency department

The majority of the patients (71.5%) received intravenous fluid whilst at the ED (Mean volume: 1394.3 mls). Proton pump inhibitor (PPI) was given in 71.5% of patients, of which 38.6% had prior peptic ulcer disease (PUD) history. 19 (59.4%) of those with a high clinical Rockall score, i.e. ≥4 received high dose PPI (i.e. 80 mg) whereas 12(37.5%) of those with the high clinical Rockall score did not receive any PPI. A small proportion of patients (18.7 and 20.3%) received Octreotide and Tranexamic acid respectively. Less than half (10, 43.5%) of those receiving Octreotide had prior h/o esophageal varices. Amongst those with h/o esophageal varices (7, 41.2%) did not receive octreotide. A minority (8, 18.6%) of those with a known history of liver disease received antibiotics in the ED. Nearly half (48.8%) of patients received blood transfusion, with 80% of them receiving 1 unit of blood. No patient received emergent endoscopy.

### Financial status and ED management (including UGI endoscopy)

We found no statistical significant between the financial status i.e. insured or non-insured patients and the ED management provided nor with the UGI endoscopy provision. (Table 4).

**Table 4:** Cross-tabulation Analysis between Financial Status and management provided

	INSURED NON-INSURED *n* = 24 *n* = 99		*p*- value
ED Management			
Intravenous fluid	18 (75.0%)	70 (70.7%)	0.676
Octreotide	4 (16.7%)	19 (19.2%)	0.776
Tranexamic acid	2 (8.3%)	23 (23.2%)	0.104
PPI	15 (62.5%)	73 (73.7%)	0.274
Antibiotics	12 (50.0%)	31 (31.3%)	0.085
Blood Transfusion	9 (37.5%)	51 (51.5%)	0.218
Receiving Endoscopy	9 (37.5%)	37 (37.4%)	0.991

### Hospital management

Upper GI endoscopy was performed in 46 (37.4%) of patients. Of these, 8(17.4%) received early Upper GI endoscopy within 24 h. All patients who received early UGI endoscopy had a low or moderate clinical Rockall score i.e. < 3 and 3–4. No patient with scores of > 4 received early UGI endoscopy. This may imply that the clinical Rockall score in this study was more of a predictor to who receives UGI endoscopy, rather than a predictor of mortality. The majority of patients received UGI endoscopy from 72 h up to 7 days post admission 15 (32.6%). None of the patients with h/o esophageal varices received early endoscopy (Table 5).

**Table 5:** Management Strategies at Emergency Department

VARIABLES	FREQUENCY (%)
ED Management	
Intravenous fluid	88 (71.5%)
Proton pump Inhibitor	88 (71.5%)
Blood Transfusion (BT)	60 (48.8%)
Antibiotics	43 (35.0%)
Tranexamic acid	25 (20.3%)
Octreotide	23 (18.7%)
Inotropy/Vasopressor support	3 (2.4%)
NGT Placement	3 (2.4%)
Emergent endoscopy	0 (0.0%)
POC results	n(n/N)
Hemoglobin < 8 g/dl (*n* = 106)*	69 (65.09%)
Urea > 7.1 mmol/l (*n* = 85)**	51 (60.0%)
Lactate > 2 meq/l (*n* = 20)	11 (55.0%)
UGI Endoscopy	46 (37.4%)
Time to Endoscopy	
> 72 h	15 (32.6%)
24–48 h	13 (28.3%)
48–72 h	10 (21.7%)
Within 24 h	8 (17.4%)

*Cut-off value based on WHO Anemia severity classification **Cut-off based on the high normal limit as stated in Glasgow-Blatchford score

### Disposition and follow up

Of those enrolled, 113 (91.8%) were admitted and 10 were discharged or eloped prior to discharge. All patients were followed up, including those discharged. Amongst those admitted, 111 (90.2%) were admitted to the wards and 2 (1.6%) to the intensive care unit (ICU). The overall 7-day mortality was 18.7%. Both patients admitted to the ICU died within 7 days. Among those admitted to the ward, 21 (18.9%) patients died within 7 days and no patients who were discharged died. 24-h mortality was 8 (34.8%).

### Predictors of 7-day mortality

Factors significantly associated with mortality in univariate analysis were age group > 40, prior H/o liver disease, active bleeding episode in the ED, provision of antibiotics in the ED and clinical Rockall score ≥ 4 (Table 6).

**Table 6:** Univariate Analysis of Factors Associated With of 7-Day Mortality

	DIED SURVIVED *n* = 23 *n* = 100		*p*- value
Age Groups			0.005
≤ 40	5 (21.7%)	54 (54.0%)	
> 40	18 (78.3%)	46 (46.0%)	
Male Sex	14 (60.9%)	73 (73.0%)	0.249
Medical History			
UGIB	5 (21.7%)	20 (20.0%)	0.852
PUD	7 (30.4%)	32 (32.0%)	0.884
Liver Disease	6 (26.1%)	6 (6.0%)	0.003
Esophageal Varices	3 (13.0%)	14 (14.0%)	0.905
UGI Endoscopy	3 (13.0%)	17 (17.0%)	0.643
Prior Hospitalization	18 (78.3%)	70 (70.0%)	0.429
Active Bleeding Episode In ED	5 (21.7%)	3 (30.0%)	0.001
ED Management			
Intravenous fluid	19 (82.6%)	69 (69.0%)	0.192
Octreotide	6 (26.1%)	17 (17.0%)	0.314
Tranexamic acid	6 (26.1%)	19 (19.0%)	0.446
PPI	15 (65.2%)	73 (73.0%)	0.456
Antibiotics	13 (56.5%)	30 (30.0%)	0.016
Blood Transfusion	13 (56.5%)	46 (46.0%)	0.410
Receiving Endoscopy	4 (17.4%)	42 (42.0%)	0.028
Clinical Rockall Score			< 0.001
< 4	8 (34.8%)	83 (83.0%)	
≥ 4	15 (65.2%)	17 (17.0%)	

In multivariate regression, age and endoscopy were independently associated with 7 day outcome: age more than 40 years was independently associated with increased mortality, whereas receiving endoscopy was associated with a reduced the risk of mortality. (Table 7).

**Table 7:** Multivariate Regression of Predictors of Mortality

	OR (95% CI)	p-value
Age > 40 years	7.0(1.7–29.2)	0.007
H/o Liver Disease	0.949(0.2–4.8)	0.949
Active Bleeding Episode in Ed	3.4(0.6–21.1)	0.185
Intravenous Fluid	1.7(0.4–7.7)	0.475
Antibiotics In ED	3.8(0.9–15.2)	0.055
Endoscopy	0.4(0.1–1.6)	0.198
Clinical Rockall Score ≥ 4*	6.4(1.8–22.8)	0.005

*Cut-off value for clinical Rockall score as per study by *Wang* et al. [19]

Variables included above are those with *p* value < 0.02 in the univariate analysis

## Discussion

UGIB in this limited income setting was associated with a high mortality rate (18.7%). One third of all deaths occurred in the first 24 h. This may be a reflection of the high disease severity seen among these patients, but also could reflect that patients did not receive guideline-adherent care. In particular, while endoscopic evaluation at any point during the hospital stay was found to be protective factor against mortality, only 37.4% of the patients received UGI endoscopy and only a handful received early UGI endoscopy i.e. within 24 h of ED presentation.

Compared with studies from HIC, patients in our study were of younger age, with the median age being 42 years [1, 13, 14, 22]. We observed that age above 40 years was an independent predictor of mortality. This has also been noted in prior studies both in HIC and LMIC [1, 5, 13, 14].

The mortality observed in our study is much higher compared to developed countries [1, 13, 14]. Factors contributing to the high observed mortality rate include; higher disease severity, disposition status and gaps in management of these patients.

Our patients had a higher disease severity in comparison to those in HIC. A large proportion of our patients had a moderate to severe clinical Rockall score (i.e. score of 3–4 and > 4) as compared to a Chinese study carried out in the ED, where most (50.6%) had a low Rockall score of 0–2 [23]. Clinical Rockall score ≥ 4 was found to be an independent predictor of higher mortality. This was also observed in several other studies (18,22). Similarly, the Glasgow-Blatchford among patients in our study was much higher compared to a British study carried out between the years 2008–2009, where the majority of patients had a GBS score ≤ 2 [24]. Severe anemia (Hb < 8 g/dl) was observed in over a half of our patients while in studies conducted in HIC, less than one-third of the patients had a Hb count suggestive of severe anemia [13, 14, 25].

As part of the ED management, majority of the patients received intravenous fluids and proton-pump inhibitor (PPI). Although international guidelines such as the European Society of Gastrointestinal Endoscopy Guidelines (ESGE) suggest use of high dose PPI followed by an hourly infusion, 19 (59.4%) patients with a high clinical Rockall score of ≥ 4 received an initial high dose PPI. It is worthy to note that none of the patients received the hourly PPI infusion as recommended in the guidelines. A knowledge gap concerning the current recommendations amongst the medical care providers maybe the plausible to this shortcoming. As per the departmental protocol, PPI should be given to all patients presenting with UGIB. This may reason out to why PPI was the first choice of drug prior to undergoing endoscopy. A small proportion of the patients received octreotide, although only half of them had indication for its use based on historical features (such esophageal varices and liver diseases) and many of those with such a history did not receive this medication. The utilization was probably limited by the availability and the cost of the drug in our setting. But the fact that some received it inappropriately suggests a knowledge gap. Antibiotic administration especially in patients with prior history of liver disease and esophageal varices was low. The National Institute of Health and Care Excellence (NICE) in the UK, emphasizes prophylactic antibiotic use in patients with suspected or confirmed variceal bleed or a history suggestive of liver disease including cirrhosis [15]. Provision of antibiotics was associated with increased incidence of mortality only in univariate analysis, probably due to confounding with the presence of liver disease which is associated with a poor outcome. Finally, despite the high proportion of patients with severe anemia, less than half of the patients received transfusion. A previous study carried out at MNH, revealed difficulties in obtaining blood for transfusion, including a general unavailability of blood, scarcity of un-cross matched blood at the blood bank along with delays in obtaining blood [26]. It is worth noting that ED management of UGIB is guided by the presence of departmental protocol for various conditions including UGIB. As such, provision of certain management may be guided by the protocol.

Only a small proportion of patients were admitted to ICU. In view of the higher disease severity in our study, a larger number of patients would be expected to be admitted into the ICU. Admission to the general ward implies that these patients were unlikely to receive the necessary aggressive care for their severity of illness; thus contributing to a high mortality rate observed. In a systematic review by *Chiu* et al it was noted that the majority of patients with a higher severity score were admitted to the ICU [27]. ICU admission for such high-risk patients has further been recommended in couple of international guidelines [15, 16]. Although the reasons for low levels of ICU admission was not determined in this study, it is presumed to be due to the limited number of ICU beds compared with the patient volume and acuity that is seen at these national referral hospitals.

UGI Endoscopic evaluation and treatment is a major cornerstone in the management of UGIB and early UGI endoscopy is associated with reduced mortality and hospital length of stay [28, 29]. Just over a third of patients (37.4%) in our study received UGI endoscopy at any time during the hospital stay; moreover only 17.5% received early endoscopy (within 24 h) and none received emergent endoscopy. The commonly used methods of hemostatic control of bleeders in our endoscopy units were hemoclipping and argon plasma coagulation (APC). Esophageal banding is usually reserved for variceal bleeders in our study setting. It is also worth noting that esophageal binding kits are not readily available in the hospital stores. Studies in HIC show that a larger proportion of their patients receive UGI endoscopy, with most receiving early UGI endoscopies [13, 14]. While literature suggests endoscopy should be done within 24 h, we found that endoscopy at any time was associated with lower mortality. This finding was statically significant in the univariate analysis but not in the multivariate regression analysis. This may be observed due to the fact that the study was not powered to evaluate the impact of endoscopy to mortality. Further studies with a larger sample size may be useful to evaluate the impact of UGI endoscopy on mortality.

## Limitations

This study was conducted at two high capacity, referral EDs and so the patient population and outcomes could be different at smaller, or lower capacity facilities. However, because these EDs receive referrals from all over the country, the patients sampled likely provide a wide representation of the Tanzanian population.

Investigations were ordered at the discretion of the physician, and thus not all patients received all tests. Some laboratory variables were not obtained for all the patients, thus this may have underestimated or overestimated the significance of these variables to the outcomes under study, including the scores such as Glasgow-Blatchford score.

The sample size for the study was estimated for our overall outcome of mortality, but not necessarily for risk factors analysis, and thus some factors not found to be significant may show statistical significance in a larger study.

## Conclusion

The mortality rate for UGIB in our setting remains substantially higher than in non-African countries. This appears to be due to the higher severity of disease in our patients, lack of ICU care, and inadequate adherence to treatment guidelines for medication and endoscopy. Some efforts may have been met with lack of resources, however further studies are needed to assess the knowledge of providers on managing patients with UGIB and familiarity with the international recommendations. Endoscopic evaluation and treatment at any point during the hospital stay still remains an independent predictor of mortality. Efforts are needed to increase the number of patients receiving endoscopic evaluation and treatment in a timely fashion in LIMC.

## Abbreviations

BP: Blood Pressure
ED: Emergency Department
EMD: Emergency Medicine Department
GBS: Glasgow-Blatchford score
HB: Hemoglobin
HIC: High Income Countries
HR: Heart rate
HVPG: Hepatic Venous Pressure Gradient
MAMC: MUHAS Academic Medical Center
MNH: Muhimbili National Hospital
MUHAS: Muhimbili University of Health and Allied Science
NVUGIB: Non-Variceal Upper Gastrointestinal Bleeding
POC: Point of Care investigations
PPI: Proton Pump Inhibitor
PUB: Peptic Ulcer Bleed
RS: Rockall score
SBP: Systolic blood pressure
UGIB: Upper Gastrointestinal Bleeding
VUGIB: Variceal Upper Gastrointestinal bleeding
